# Longitudinal course of neurofilament light chain levels in amyotrophic lateral sclerosis—insights from a completed randomized controlled trial with rasagiline

**DOI:** 10.1111/ene.16154

**Published:** 2023-11-17

**Authors:** Simon Witzel, Jeffrey M. Statland, Petra Steinacker, Markus Otto, Johannes Dorst, Joachim Schuster, Richard J. Barohn, Albert C. Ludolph

**Affiliations:** ^1^ Department of Neurology Ulm University Ulm Germany; ^2^ University of Kansas Medical Center Kansas City Kansas USA; ^3^ Department of Neurology University of Halle Halle (Saale) Germany; ^4^ German Center for Neurodegenerative Diseases (DZNE) Ulm Germany; ^5^ School of Medicine, NextGen Precision Health, University of Missouri Columbia Missouri USA

**Keywords:** amyotrophic lateral sclerosis, clinical trial design, neurofilament light, rasagiline

## Abstract

**Background and purpose:**

Rasagiline might be disease modifying in patients with amyotrophic lateral sclerosis (ALS). The aim was to evaluate the effect of rasagiline 2 mg/day on neurofilament light chain (NfL), a prognostic biomarker in ALS.

**Methods:**

In 65 patients with ALS randomized in a 3:1 ratio to rasagiline 2 mg/day (*n* = 48) or placebo (*n* = 17) in a completed randomized controlled multicentre trial, NfL levels in plasma were measured at baseline, month 6 and month 12. Longitudinal changes in NfL levels were evaluated regarding treatment and clinical parameters.

**Results:**

Baseline NfL levels did not differ between the study arms and correlated with disease progression rates both pre‐baseline (*r* = 0.64, *p* < 0.001) and during the study (*r* = 0.61, *p* < 0.001). NfL measured at months 6 and 12 did not change significantly from baseline in both arms, with a median individual NfL change of +1.4 pg/mL (interquartile range [IQR] −5.6, 14.2) across all follow‐up time points. However, a significant difference in NfL change at month 12 was observed between patients with high and low NfL baseline levels treated with rasagiline (high [*n* = 13], −6.9 pg/mL, IQR −20.4, 6.0; low [*n* = 18], +5.9 pg/mL, IQR −1.4, 19.7; *p* = 0.025). Additionally, generally higher longitudinal NfL variability was observed in patients with high baseline levels, whereas disease progression rates and disease duration at baseline had no impact on the longitudinal NfL course.

**Conclusion:**

Post hoc NfL measurements in completed clinical trials are helpful in interpreting NfL data from ongoing and future interventional trials and could provide hypothesis‐generating complementary insights. Further studies are warranted to ultimately differentiate NfL response to treatment from other factors.

## INTRODUCTION

Inhibiting MAO‐B rasagiline reduces dopamine and serotonin catabolism, thereby increasing dopamine and serotonin availability for neurotransmission. In amyotrophic lateral sclerosis (ALS), prominent pathological involvement of dopaminergic [1, 2] and serotonergic neurons [3, 4] has been described. Moreover, rasagiline also possesses antiapoptotic and antioxidative properties [5, 6, 7], which could contribute to ALS pathogenesis [8]. In the SOD1‐G93A ALS mouse model, rasagiline showed a dose‐dependent therapeutic effect on motor function and prolonged survival by about 20% [9]. Evidence in humans comes from Parkinson's disease, where neuroprotective effects were first observed in the TEMPO trial [10] and later reproduced in the ADAGIO trial [11, 12] in patients in the 1 mg/day rasagiline arm, but, interestingly, not the 2 mg/day arm. However, it is unknown which mechanism of action of rasagiline might be associated with a neuroprotective effect and whether the results from Parkinson's disease could be translated to ALS.

Rasagiline has been evaluated as a disease‐modifying drug in randomized, placebo‐controlled trials (RCTs) in patients with ALS in a dosage of 1 mg/day [13] and 2 mg/day [14]. In the RCT with 1 mg/day by Ludolph et al. [13], including 252 participants randomized in a 1:1 ratio, the primary end‐point was survival time during an intervention period of 18 months; in the 2 mg/day RCT by Statland et al. [14], including 80 participants randomized in a 3:1 ratio, the primary end‐point was the average slope of decline on the ALS Functional Rating Scale Revised (ALSFRS‐R) after 12 months. Neither trial observed a significant disease‐modifying effect of rasagiline on the primary outcome parameter in the intention‐to‐treat analysis.

However, a post hoc analysis conducted in the 1 mg/day trial indicated a benefit on survival after 6 months in the whole study population (hazard ratio 0.32; 95% confidence interval [CI] 0.11–0.86; *p* = 0.0178) and a significant effect on disease progression (median loss of ALSFRS‐R points/month: rasagiline 1.03, interquartile range [IQR] 0.65, 1.66, vs. placebo 1.51, IQR 1.16, 2.35; *p* = 0.0051) in the subpopulation of patients with intermediate to fast disease progression (ALSFRS‐R slope greater than 0.5 points/month). A similar post hoc analysis of ALSFRS‐R scores in the 2 mg/day trial was not possible due to (1) a considerably smaller sample size, (2) 3:1 randomization into treatment and placebo groups, (3) a relatively high dropout rate and (4) incomplete matching of baseline (BL) characteristics between study participants and historical controls, which were used to fill up the placebo group [14]. However, a strength of the 2 mg trial was a standardized collection of blood samples enabling further analyses of potential disease‐modifying effects of rasagiline by longitudinal measurements of prognostic biomarkers. In contrast to the 2 mg/day trial, there was no biosampling in the 1 mg/day trial.

Neurofilament light chain (NfL) is a marker for axonal loss and is being established as a diagnostic and prognostic biomarker in ALS [15]. A robust correlation of NfL blood levels with disease progression and survival in ALS has repeatedly been reported [16, 17, 18, 19, 20, 21, 22]. Given these correlations and a relative longitudinal stability in individual patients over trial‐relevant periods, NfL is regarded as a promising biomarker end‐point for clinical trials. Biomarker data from recent interventional trials indicate the utility of NfL as a potential surrogate marker for clinical outcomes [23, 24].

In this study, the longitudinal course of NfL levels was assessed in blood samples collected during an RCT with 2 mg rasagiline in ALS. The aim was to achieve two primary objectives: (1) to evaluate the correlation between NfL BL levels and clinical parameters and (2) to investigate the individual longitudinal NfL course to determine whether potential disease‐modifying effects of rasagiline, particularly in patients with intermediate to fast disease progression, could be reflected in a reduction of NfL levels.

## METHODS

### Study design and biomarker measurement

A biomarker study was conducted followed by correlation with clinical parameters using biosamples and clinical data collected throughout the randomized, double‐blind, placebo‐controlled trial with rasagiline 2 mg/day in patients with ALS registered at clinicaltrials.gov (NCT01786603) and published previously by Statland *et al*. [14]. Detailed information on trial methods can be found in that publication. In brief, the main trial inclusion criteria were age between 21 and 80 years, probable (including laboratory supported) or definite ALS by El Escorial criteria [25], relative slow or forced vital capacity (FVC) of ≥75%, and onset of symptoms within 2 years before enrolment. Participants (*n* = 80) were randomized in a 3:1 ratio to 2 mg/day rasagiline or placebo. The intervention lasted 12 months. The primary outcome parameter was the slope of decline of the ALSFRS‐R, and the secondary outcome parameters were changes in slow vital capacity, the global ALS Quality of Life score and invasive‐ventilation‐free survival.

The RCT (NCT01786603), biomarker sampling and analysis were approved by the institutional review board of the University of Kansas Medical Centre (approval ID MODCR00003230). Written informed consent was obtained from all participants, in accordance with the Declaration of Helsinki and the principles of good clinical practice.

Biosamples and clinical data were collected at BL (time of randomization), month 6 and month 12. Clinical BL data incorporated sex, age, disease duration since onset of symptoms, riluzole use, bulbar versus non‐bulbar onset of symptoms, ALSFRS‐R score and relative FVC. Clinical follow‐up data included ALSFRS‐R scores and survival; survival status was followed up until July 2016. Disease progression rates were defined as the slope of the decline of the ALSFRS‐R score (in score points per month). Disease progression rates pre‐baseline were computed using the formula

ALSFRS‐R pre‐slope = (ALSFRS‐R at BL – 48)/disease duration since symptom onset in months

Disease progression rates during the study were calculated using the formula

ALSFRS‐R study slope = (last ALSFRS‐R score – ALSFRS‐R score at BL)/months from BL to the last score

Blood sample collection, pre‐analytical phase, and storage were defined by standard operating procedures. NfL levels were measured in plasma with the single molecule array (Simoa) platform provided by Quanterix using a commercially available kit (NF‐light, Quanterix) with single measurements (coefficient of variation <15%) [17].

### Statistical analysis

Items were tested for normal distribution using the Kolmogorov–Smirnov test. Mean and standard deviation are reported for values with normal distribution; otherwise, median with IQR (quartile 1, quartile 3). A two‐sided *t* test for either independent or related samples was used to compare normally distributed variables; in not normally distributed variables, the Mann–Whitney *U* test (independent samples) and the Wilcoxon signed‐rank test (related samples) were used to compare groups. Categorical variables were compared using the *χ* ^2^ test. The correlation of NfL levels with disease progression rates was analysed using a linear regression model; outliers were defined as values outside of 3 standard deviations and were excluded from linear regression analysis: this affected 2 data points in the correlation of NfL levels with disease progression rates pre‐baseline and no data points in the correlation of NfL levels with disease progression rates during the study. All tests were performed at a level of significance of *p* = 0.05. As an explorative study, no adjustment for multiple testing was made. Accordingly, all results were interpreted as hypothesis‐generating rather than proof of a specific hypothesis. Missing data were handled via pairwise deletion; in variables with less than 95% of data points available, the exact number of analysed cases is reported. Analyses were performed in the entire dataset to compare treatment groups (placebo vs. rasagiline) and additionally in subgroups with high (above median) and low (below median) BL NfL levels.

To evaluate the individual longitudinal NfL variability, the median and the 10th, 25th, 75th and 90th percentile of the absolute and relative change of follow‐up NfL values were analysed from a patient's BL NfL value; the analysis was done for all patients and in the subgroups with high and low BL NfL levels. Additionally, the individual longitudinal NfL change in dependence from the BL ALSFRS‐R scores and disease duration at BL was analysed graphically and via linear regression analysis.

SPSS version 28.0.1 was used for statistical analysis.

## RESULTS

Baseline blood samples were available in 65 out of 80 patients: *n* = 17 in patients randomized to placebo, and *n* = 48 in patients randomized to rasagiline. Clinical parameters and NfL levels at BL did not significantly differ between the treatment groups (Table 1).

**TABLE 1:** Baseline characteristics.

Variable	Total	Placebo arm	Rasagiline arm	*p* value
Number of participants	65	17	48	–
Sex, *n* (%)	Female 23 (35%)/male 42 (65%)	Female 5 (29%)/male 12 (71%)	Female 18 (38%)/male 30 (62%)	0.77
Age, years, mean (SD)	57.8 (±9.5)	56.2 (±8.1)	58.3 (±10.0)	0.44
Disease duration, months, median (IQR)	18 (12, 21)	18 (12, 22)	17 (10, 20)	0.56
Riluzole use, *n* (%)	55 (85%)	14 (82%)	41 (85%)	0.52
Bulbar onset, *n* (%)	8 (12%)	2 (12%)	6 (13%)	0.65
ALSFRS‐R, points, median (IQR)	39 (34, 42)	35 (30, 43)	39 (35, 42)	0.11
ALSFRS‐R pre‐slope, points/month, median (IQR)	−0.57 (−0,83, −0,38)	−0.67 (−1.24, −0.49)	−0.53 (−0.76, −0.37)	0.24
FVC, %, mean (SD)	93.5 (±11.6)	93.6 (±9.4)	93.5 (±12.4)	0.97
NfL, pg/mL, median (IQR)	51.8 (35.5, 85.9)	51.8 (41.8, 81.5)	52.0 (32.6, 90.4)	0.85

Abbreviations: ALSFRS‐R, Amyotrophic Lateral Sclerosis Functional Rating Scale Revised; FVC, forced vital capacity; IQR, interquartile range; NfL, plasma neurofilament light chain.

**FIGURE 1:**
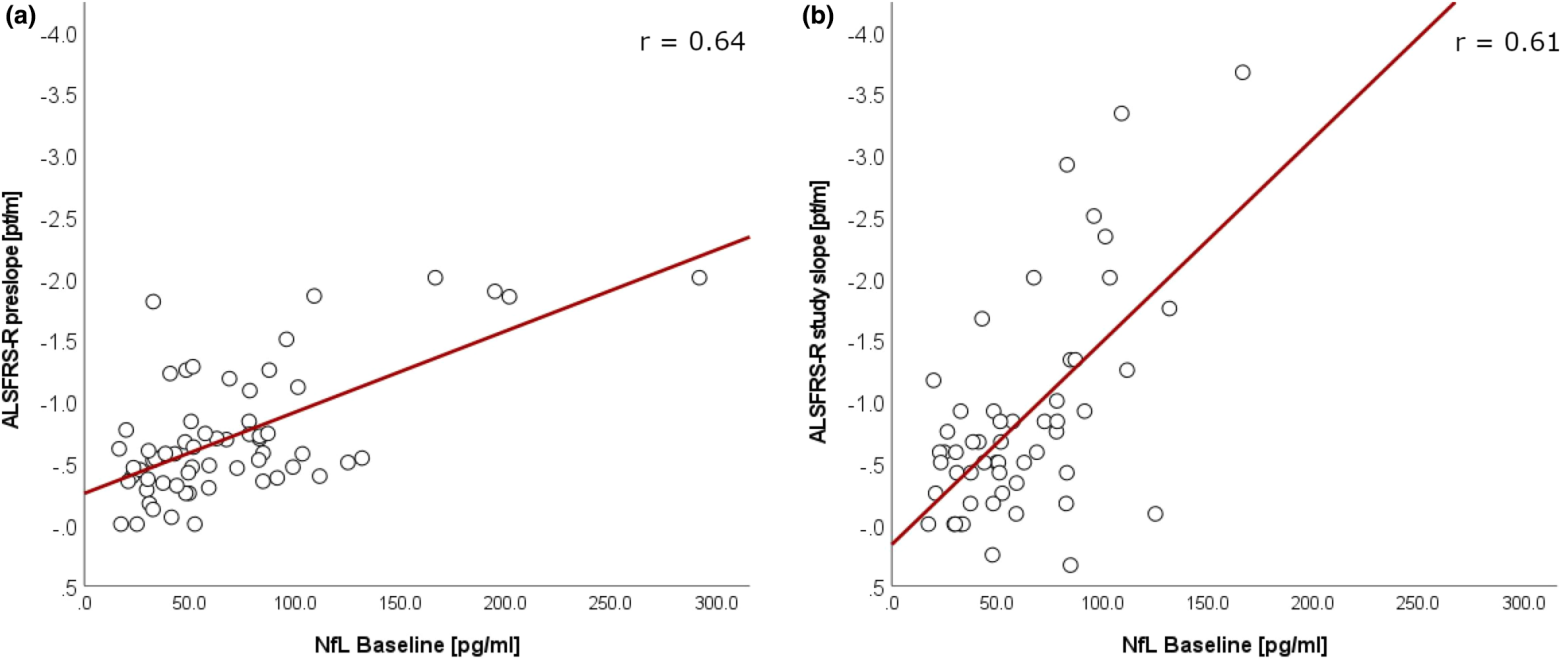
Correlation between baseline NfL levels and disease progression. Scatter plots showing the correlation between NfL baseline levels and the slope of the ALSFRS‐R decrease before the study ((a) pre‐slope) and throughout the study ((b) study slope) in all participants. ALSFRS‐R, Amyotrophic Lateral Sclerosis Functional Rating Scale Revised; NfL, plasma neurofilament light chain.

The median BL NfL level was 51.8 pg/mL amongst all patients. BL NfL levels significantly correlated with disease progression rates pre‐baseline (*r* = 0.64, *p* < 0.001) and disease progression rates during the study (*r* = 0.61, *p* < 0.001) (Figure 1).

Patients with high BL NfL levels (NfL > median), compared with patients with low BL NfL levels (NfL ≤ median), showed significantly faster disease progression rates pre‐baseline (high BL NfL, −0.69 points/month, IQR −1.16, −0.48; low BL NfL, −0.46 points/month, IQR −0.65, −0.30; *p* = 0.009) and during the study (high BL NfL, −0.88 points/month, IQR −2.00, −0.40; low BL NfL, −0.50 points/month, IQR −0.71, −0.17; *p* = 0.007). All other clinical characteristics did not differ significantly (Table 2).

**TABLE 2:** Clinical characteristics amongst patients with low and high baseline NfL levels.

Variable	Low baseline NfL	High baseline NfL	*p* value
Number of participants	33	32	–
NfL, pg/mL, median (IQR)	37.3 (28.0, 48.0)	85.9 (73.9, 111.0)	
Sex, *n* (%)	Female 14 (42%)/male 19 (58%)	Female 9 (28%)/male 23 (72%)	0.17
Age, years, mean (SD)	57.2 (±9.1)	58.3 (±10.0)	0.66
Disease duration, months, median (IQR)	18 (13, 22)	15 (9, 20)	0.09
Riluzole use, *n* (%)	28 (85%)	27 (85%)	0.61
Bulbar onset, *n* (%)	3 (9%)	5 (16%)	0.37
ALSFRS‐R, points, median (IQR)	39 (35, 44)	38 (33, 42)	0.17
ALSFRS‐R pre‐slope, points/month, median (IQR)	−0.46 (−0.65, −0.30)	−0.69 (−1.16, −0.48)	**0.009**
ALSFRS‐R study slope, points/month, median (IQR)	−0.50 (−0.71, −0.17)	−0.88 (−2.00, −0.40)	**0.007**
FVC, %, mean (SD)	91.3 (±10.7)	95.7 (±12.3)	0.13
FVC change, %/month, median (IQR)	−1.3 (−2.0, −0.1)	−2.0 (−4.9, −0.9)	0.07

Abbreviations: ALSFRS‐R, Amyotrophic Lateral Sclerosis Functional Rating Scale Revised; FVC, forced vital capacity; IQR, interquartile range; NfL, plasma neurofilament light chain.

Although the number of deceased patients was low throughout the follow‐up period, a significantly shorter survival time was observed amongst patients with high BL‐NfL levels (events: *n* = 6; mean survival time 12.3 months, 95% CI 11.2–13.4) compared with those with low BL‐NfL levels (events: *n* = 2; mean survival time 15.9 months, 95% CI 15.4–16.3; log‐rank *p* = 0.03).

**FIGURE 2:**
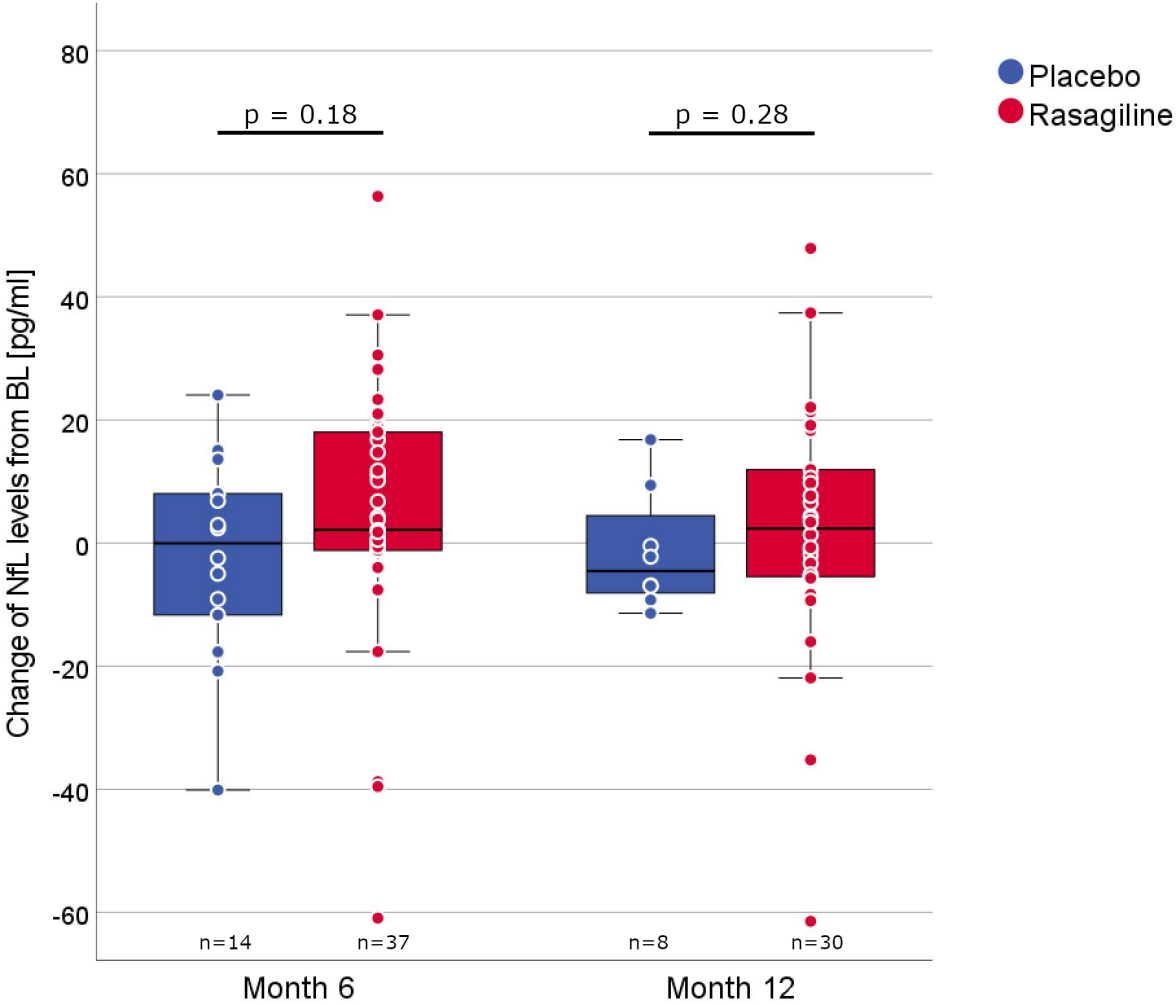
Change of NfL levels. The change of NfL levels between baseline and months 6 and 12 displayed with box plots (median, central line; IQR, boxes; 1.5 × IQR, whiskers) overlaid with dots for single patients. *p* values refer to group comparisons using the Mann–Whitney *U* test. BL, baseline; IQR, interquartile range; NfL, plasma neurofilament light chain.

The longitudinal analysis did not show a significant change in NfL levels from BL to month 6 (*n* = 51, +2.1 pg/mL, IQR −4.2, 15.1; *p* = 0.15) or month 12 (*n* = 38, −0.57 pg/mL, IQR −6.9, 11.0; *p* = 0.51) in the entire cohort. Likewise, no significant changes were observed in patients receiving placebo (month 6, *n* = 14, ±0.0 pg/mL, IQR −13.2, +9.4, *p* = 0.73; month 12, *n* = 8, −4.5 pg/mL, IQR −8.7, +6.9, *p* = 0.57) or rasagiline (month 6, *n* = 37, +2.1 pg/mL, IQR −2.6, +18.5, *p* = 0.07; month 12, *n* = 30, +2.4 pg/mL, IQR −5.5, +13.5, *p* = 0.35). Additionally, no significant difference was observed in changes in NfL levels between the treatment groups at months 6 and 12 compared with BL (Figure 2).

Study dropout before the first follow‐up at month 6 occurred in 10 patients (*n* = 3 [18%] in controls; *n* = 7 [15%] in the rasagiline arm). Although no significant difference in NfL BL levels was observed between patients with early dropout compared with patients who completed the study or dropped out after month 6 (*p* = 0.21), it was observed that, amongst patients with NfL BL values above the 90th percentile (128 pg/mL), four dropped out before month 6, whereas only two completed the first follow‐up and only one the entire study.

**FIGURE 3:**
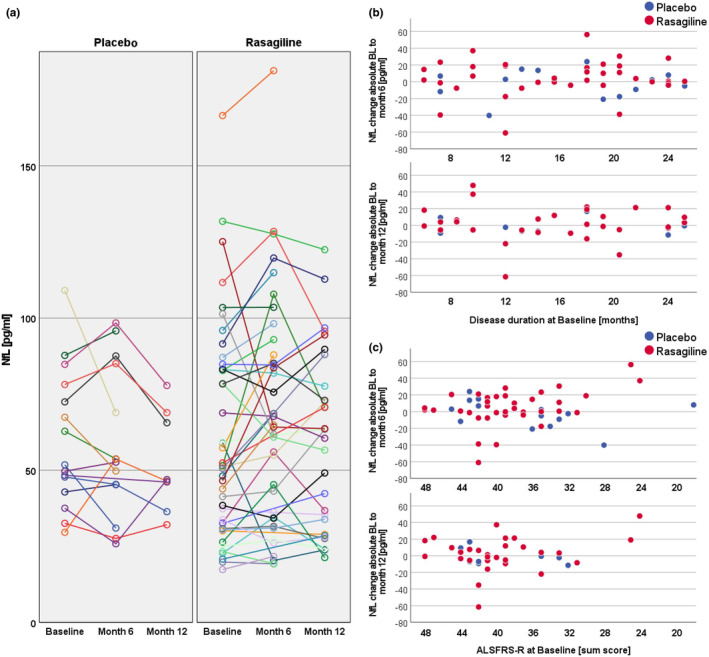
Individual longitudinal change in NfL levels. The panels show the change in NfL levels in individual patients. (a) Spaghetti plots, visualizing the course of NfL levels in each patient. (b) The individual change of NfL from baseline values across disease duration at baseline at months 6 (upper plot) and 12 (lower plot). (c) The individual change of NfL from baseline values across the ALSFRS‐R sum score at baseline at months 6 (upper plot) and 12 (lower plot). ALSFRS, Amyotrophic Lateral Sclerosis Functional Rating Scale Revised; BL, baseline; NfL, plasma neurofilament light chain.

The individual median longitudinal NfL change was close to zero (+1.4 pg/mL), with 80% of the individual deviation from BL values found in a range between −17.6 pg/mL (10th percentile) and +22.1 pg/mL (90th percentile), and half of the values even in a narrow range between −5.6 pg/mL (25th percentile) and +14.2 pg/mL (75th percentile). This corresponded to a median relative change of +4.3% (IQR −10.8%, +24.9%). Higher absolute individual deviations were observed in patients with high BL NfL values (percentiles: 10th −39.4 pg/mL; 25th −12.7 pg/mL; median −1.2 pg/mL; 75th +11.5 pg/mL, 90th +18.9 pg/mL) compared with patients with low BL NfL values (percentiles: 10th −5.9 pg/mL; 25th −2.1 pg/mL; median −2.7 pg/mL; 75th +18.7 pg/mL, 90th +25.4 pg/mL). The median relative change from BL levels was −1.7% (IQR −14.4%, 12.5%) amongst patients with high BL levels and +6.8% (IQR −4.9%, +42.3%) amongst patients with low BL levels. No significant correlation was observed between BL ALSFRS‐R scores (linear regression at month 6, *p* = 0.25; at month 12, *p* = 0.28) or BL disease duration (linear regression at month 6, *p* = 0.63; at month 12, *p* = 0.85) with the individual NfL change (Figure 3).

**FIGURE 4:**
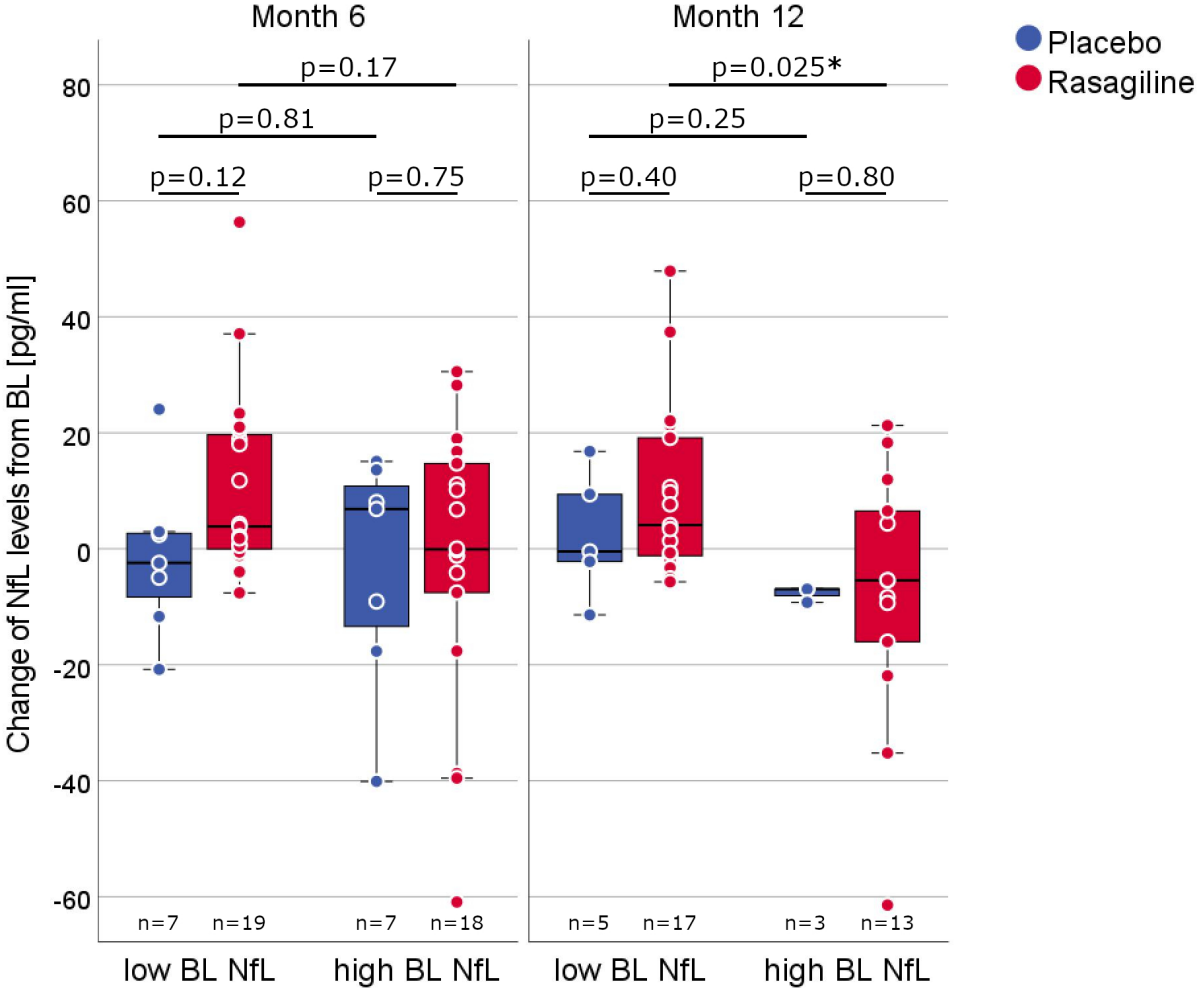
Change of NfL levels in subgroups with high and low NfL baseline levels. The change of NfL levels between baseline and months 6 (left panel) and 12 (right panel) in the subgroups with high (above median) and low (below median) NfL levels at baseline shown as box plots (median, central line; IQR, boxes; 1.5 × IQR, whiskers) overlaid with dots for single patients. *p* values refer to group comparisons using the Mann–Whitney *U* test. BL, baseline; NfL, plasma neurofilament light chain.

The subgroup analysis in patients with high and low BL NfL levels showed no significant differences between the treatment groups at months 6 and 12. However, in patients treated with rasagiline, a significant difference was observed in the change of NfL levels from BL to month 12 between patients with high and low BL NfL levels (high, *n* = 13, −6.9 pg/mL, IQR −20.4, 6.0; low, *n* = 18, +5.9 pg/mL, IQR −1.4, 19.7; *p* = 0.025); this was not observed at month 6. In patients who received placebo, no significant differences were observed between the high and low BL NfL subgroups (Figure 4).

## DISCUSSION

Improved methods for patient stratification and a redefinition of outcome measures are considered key aspects of trial design evolution in ALS [26, 27, 28]. Recent phase II and III trials have shown that NfL lowering corresponds to beneficial clinical outcomes, suggesting its potential as a surrogate biomarker for disease progression and survival [23, 24, 29]. Notably, the significant longitudinal NfL decrease observed in patients treated with tofersen, an antisense oligonucleotide targeting SOD protein in SOD1‐associated ALS, preceded the clinical outcome and played a decisive role in its Food and Drug Administration approval [30]. Consequently, conducting NfL measurements in completed clinical trials provides complementary data that may shed new light on negative trial outcomes. Even in the absence of a biomarker signal, additional NfL data from controlled studies remain crucial to better understand the biomarker's correlation with clinical parameters and differentiate natural variations and other factors from treatment effects.

The present study demonstrates a robust correlation between BL NfL levels and ALSFRS‐R‐based disease progression rates. Additionally, patients with low and high BL NfL levels exhibited significantly different survival outcomes. Both findings are consistent with previous studies investigating the prognostic value of NfL blood levels in ALS [16, 17, 18, 19, 20, 21, 31]. Notably, patients with high BL NfL levels also displayed a trend for a faster decline in FVC, a measure of respiratory function in ALS, warranting further evaluation in larger cohorts. The prognostic significance of BL NfL levels indicates a utility for biomarker‐based patient stratification and supports its role as a prognostic biomarker for disease progression and survival.

In the entire cohort of rasagiline‐treated patients, a significant longitudinal NfL lowering was not observed. However, on comparing patients with high BL NfL levels with those with low BL levels, a difference in NfL level changes was found at month 12 in the rasagiline arm. Clinically, patients with high BL NfL levels had significantly faster disease progression before and during the study, whilst demographic characteristics and ALS‐specific factors such as the proportion of patients with bulbar disease onset, ALSFRS‐R sum score at BL, riluzole use and respiratory function did not differ. Although not statistically significant, patients with high NfL levels often had shorter disease duration at BL. Unfortunately, only three patients with high BL NfL values in the placebo group completed the 12‐month follow‐up, making a reliable comparison between rasagiline and placebo unfeasible. Previous studies offer few data on the course of NfL levels in ALS patients with high BL values: in the VALOR study investigating tofersen in SOD1‐associated ALS, patients with high BL NfL levels receiving placebo (*n* = 21) showed a 20% increase in NfL levels after 28 weeks [24]. Additionally, the published individual trajectories of NfL blood levels in ALS patients collected in previous studies by our group [16, 23] showed predominantly stable or increasing levels in patients with high BL NfL levels across different cohorts.

Given the potential bias introduced by high dropout rates, the relatively small number of patients who completed the 12‐month follow‐up and the lack of a direct comparison with a placebo group, the interpretation of the NfL finding in the rasagiline arm remains hypothetical. One possibility is that it could reflect a biomarker response to rasagiline in fast‐progressing patients as indicated by a beneficial clinical effect of rasagiline observed in fast‐progressing patients in a post hoc analysis of the 1 mg rasagiline trial [13]. However, an alternative explanation might be a coincidental finding within a higher longitudinal NfL variability in patients with high NfL levels, as larger absolute longitudinal NfL variations were generally observed in patients with high BL values when all longitudinal measurements were examined regardless of a patient's treatment arm.

In contrast to the higher variability observed in association with high BL NfL levels, further analyses of the entire cohort revealed that ALSFRS‐R scores and disease duration at BL had no significant impact on the NfL course during the study period. These findings align with previous work from our group [16] and are important for interventional studies, as they support longitudinal NfL stability in disease stages and time frames most relevant for interventional trials. Considering that almost all ongoing therapy studies in ALS include neurofilaments as end‐points, our study might provide helpful neurofilament reference data and new insights into the longitudinal course under specific preconditions.

The main limitation of our study is the relatively small number of patients in the placebo group, a consequence of the 3:1 randomization and high dropout rates. The absence of follow‐up information for patients who dropped out before the first follow‐up is a significant concern not only in our study but also in other ALS trials, as it compromises the study's power and introduces potential bias. Although there are various reasons for study dropout, a rapid decline in functional status linked to increasing immobility appears to be a primary factor leading ALS patients to discontinue study participation. Notably, it was observed that patients above the 90th percentile of NfL BL levels were more likely to drop out early during the study, suggesting that specifying inclusion criteria in future trials could help mitigate the probability of early study dropout.

In the context of the present study, it is worth looking at the obstacles to further establishing neurofilaments as neuronal biomarkers in general and in ALS in particular. Internationally standardized methods for measuring neurofilaments, reference values for various neurological conditions, and accurate quantification of confounding factors are remaining challenges to improve reliability and comparability. In ALS, the diagnostic specificity of neurofilaments is inherently limited by their nature as a non‐specific marker of axonal loss, but their potential for prognostication and monitoring of treatment response is very promising and may prove essential to address the challenge of highly heterogeneous disease progression. Population‐based neurofilament reference data will be helpful in overcoming selection bias in hospital‐based research and clinical trials and will be critical in translating findings to a broader ALS population encountered in real‐world settings. Ultimately, translating treatment‐related neurofilament lowering into clinical outcomes at the patient level will be essential for individualized medicine once multiple effective treatment options are available.

## CONCLUSION

Although our study could not show a significant longitudinal NfL lowering in the entire cohort of rasagiline‐treated patients, it demonstrates that post hoc NfL measurements in completed clinical trials provide complementary data, which (1) can offer additional hypothesis‐generating insights into negative trials and (2) can help to interpret longitudinal NfL data from ongoing and future interventional studies. Further research is warranted to understand the implications of longitudinal changes in NfL levels and to differentiate when these changes reflect a disease‐modifying effect and when they are attributable to other factors.

## AUTHOR CONTRIBUTIONS

**Simon Witzle:** Conceptualization; writing – original draft; visualization; formal analysis; data curation. **Jeffrey Statland:** Conceptualization; investigation; data curation; writing – review and editing; funding acquisition; project administration; resources; validation. **Petra Steinacker:** Investigation; writing – review and editing. **Markus Otto:** Conceptualization; supervision; investigation; writing – review and editing; resources. **Johannes Dorst:** Conceptualization; supervision; writing – review and editing; validation. **Joachim Schuster:** Conceptualization; project administration; writing – review and editing; validation. **Richard J. Barohn:** Investigation; funding acquisition; project administration; supervision; writing – review and editing; resources. **Albert Christian Ludolph:** Conceptualization; project administration; supervision; resources; writing – review and editing.

## FUNDING INFORMATION

No funding was received towards the present study. Fundings received for the original randomized controlled trial (NCT01786603): US Food and Drug Administration Orphan Products Division (RO1 FD003739 to H.M.W., R.H.S. and R.J.B.; P30 AG035982 to H.M.W. and R.H.S.); NCATS grant awarded to the University of Kansas Medical Centre for Frontiers: The Heartland Institute for Clinical and Translational Research (CTSA grants UL1TR000001 and KL2TR000119 to J.M.S.). The funding sources had no influence on conceptualization, analysis or interpretation of the present study.

## CONFLICT OF INTEREST STATEMENT

All authors declare no support from any organization for the submitted work; ACL has received honoraria from TEVA Pharmaceutical Industries outside of the submitted work. All other authors declare no financial relationships with any organizations that might have an interest in the submitted work in the previous 3 years; no other relationships or activities that could appear to have influenced the submitted work.

## Data Availability

Data will be shared with researchers upon reasonable request.
